# Characterization of *APOE* Christchurch carriers in 455,306 UK Biobank participants

**DOI:** 10.1186/s13024-023-00684-7

**Published:** 2023-11-28

**Authors:** Karen Y. He, Ekaterina A. Khramtsova, Alfredo Cabrera-Socorro, Yanfei Zhang, Shuwei Li, Brice A. J. Sarver, Bart Smets, Qingqin S. Li, Louis De Muynck, Antonio R. Parrado, Simon Lovestone, Mary Helen Black

**Affiliations:** 1 Population Analytics & Insights, Data Science Analytics & Insights, Janssen R&D, Spring House, PA USA; 2 Neuroscience Therapeutic Area, Janssen R&D, Beerse, Belgium; 3 Neuroscience Data Science, Janssen R&D, Beerse, Belgium; 4 Neuroscience Data Science, Janssen R&D, Titusville, NJ USA

**Keywords:** APOE-Christchurch variant, Alzheimer’s disease, Rare variants

**To the editor**

The *APOE* gene is a known genetic risk factor for neurodegeneration and cardiovascular disease (CVD) [1, 2]. Beyond the known effects of *APOE* ε2 and APOE ε4, several rare and protective *APOE* variants (R154S Christchurch (APOECh), V236E Jacksonville, and R251G) have been identified recently [3, 4]. The ultra-rare APOECh mutation (NM_000041.4(APOE):c.460C > A (p.Arg154Ser)), also known as p.Arg136Ser or R136S in previous publications [5, 6], has been hypothesized to protect from *PSEN1*-based autosomal dominant Alzheimer’s disease (AD) [5]. The protective effect of APOECh was observed for a single homozygous individual [5] but has been debated for heterozygous individuals [7].

In addition to unraveling the molecular mechanisms by which *APOE* genetic variants affect lipid biology and neuropathological features including amyloid plaques and tau tangles, immune response, vascular integrity and function, and other AD-related pathways [3, 4], it is important to understand what other phenotypic manifestations they can cause. To date, few studies have reported on the effect of APOECh due to limited number of carriers in the population [5, 6, 8].

Using UK Biobank whole exome (WES; *N* = 454,756) and whole genome (WGS; *N* = 141,948) sequences, we identified 37 heterozygous individuals (27 females and 10 males; 36 Europeans, 1 admixed American), resulting in APOECh allele frequency of 0.004% (Supplementary Methods, Supplementary Table 1, Additional File 1) and evaluated 19 binary traits on neurological disorders, cardiovascular disorders, and medication use, and 80 quantitative traits, including blood and urine-based biomarkers (Supplementary Fig. 1 and Supplementary Tables 2–4, Additional File 1).

It is important to consider the effect of APOECh in the context of other AD risk or protective variants. We find that APOECh carriers are enriched (*p* = 0.001) for the ε3/ε3 genotype; five carriers (13.9%) are heterozygous for *APOE* ε4, 30 (83.3%) are homozygous for *APOE* ε3, and one (2.8%) has an ε2/ε3 genotype (Supplementary Table 5, Additional File 1). Several APOECh carriers also carry single nucleotide risk alleles in the *GRN*, *SORT1*, and *APBB2* genes, but none had risk mutations in the highly penetrant genes (i.e., *APP*, *PSEN1*, *PSEN2*) associated with early-onset AD (Supplementary Table 6, Additional File 1). While only one carrier had one copy of the protective *APOE* ε2 allele, none of them had the protective V236E Jacksonville and R251G variants (Supplementary Table 6, Additional File 1).

We did not detect any significant difference between carriers and noncarriers for the binary traits assessed (Supplementary Table 7, Additional File 1). Among the heterozygous carriers (age: 56.62–82.06, median: 68.63), none have developed AD or mild cognitive impairment by March 2022 (data freeze used), including 4 individuals with parental history of AD (Supplementary Table 1, Additional File 1). While this may suggest that the APOECh carriers are protected, continued follow-up as the cohort ages is necessary. Interestingly, the carriers showed a decreased genetic risk measured by polygenic risk score (PRS) for Alzheimer’s disease (*p* = 0.02), which cannot be attributed to APOECh, as this rare variant is not included in AD genome-wide association studies and PRS calculations (Supplementary Methods and Supplementary Table 8, Additional File 1).

Among non-lipid blood biomarkers and hematological traits, a few traits showed differences at a nominal level (*p* < 0.05) but were not significant after accounting for multiple testing of 22 non-lipid blood biomarkers or 31 hematological traits (Supplementary Table 8 and Supplementary Figs. 2–5, Additional File 1). Importantly, among blood-based lipid biomarkers measured at baseline (Table 1 and Supplementary Fig. 6, Additional File 1), both unadjusted and adjusted for self-reported statin use at the time of recruitment, apolipoprotein B (apoB) levels were significantly lower in carriers compared to noncarriers (*p* = 0.004 and *p* = 0.036, respectively). While statin adjustment slightly attenuated the difference in apoB levels, the significant difference persisted, suggesting that lower apoB may be a characteristic of APOECh carriers and having lower apoB may be protective against AD. We also found APOECh carriers to have lower median apoB/apoA1 ratio vs. noncarriers (*p* = 0.047), which together with lower apoB suggests protection from major adverse cardiovascular event (MACE) despite small sample size to directly detect this for ICD-coded events. Given the role of apoB in major vascular diseases, we also assessed for differences in cerebral amyloid angiopathy or vascular dementia diagnoses among carriers and noncarriers, but found no difference (Supplementary Table 7, Additional File 1), most likely due to small sample size.

**Table 1:** Blood-based lipid biomarkers of APOECh carriers and noncarriers

	EUR Carriers (*N*=36)		EUR Noncarriers (*N*=129,240)		KS test
	Min, Max	Median	Min, Max	Median	
Apolipoprotein A	1.03-2.12	1.64	0.52-2.50	1.55	D=0.19 P-value=0.239
Apolipoprotein B	0.65-1.37	0.88	0.40-1.99	1.02	D=0.31 P-value=0.004
Apolipoprotein B (statin adjusted)	0.65-1.37	0.94	0.40-2.76	1.06	D=0.25 P-value=0.036
C reactive protein	0.09-16.48	1.12	0.08-78.26	1.32	D=0.12 P-value=0.662
Cholesterol	3.82-7.83	5.32	1.71-12.50	5.73	D=0.18 P-value=0.263
Cholesterol (statin adjusted)	3.82-7.83	5.80	1.88-15.56	5.91	D=0.15 P-value=0.427
High-density lipoprotein	0.80-2.05	1.56	0.23-4.19	1.47	D=0.2 P-value=0.225
Low-density lipoprotein	2.19-5.06	3.25	0.75-8.86	3.55	D=0.23 P-value=0.072
Low-density lipoprotein (statin adjusted)	2.23-5.06	3.36	0.80-12.96	3.70	D=0.21 P-value=0.119
Lipoprotein A	4.10-178.95	22.00	3.80-189.00	20.50	D=0.11 P-value=0.838
Triglycerides	0.79-6.31	1.71	0.23-11.15	1.42	D=0.16 P-value=0.329

Summary statistics of lipid biomarkers among European APOECh carriers and matched noncarriers. A two-sided Kolmogorov-Smirnov (KS) test was used to determine whether the measurements for a given lipid biomarker follow the same distribution among carriers and noncarriers

Higher levels of plasma apoB are associated with AD or cognitive decline [9, 10] and a recent study has demonstrated that cerebrospinal fluid apoB levels are correlated with tau pathology in pre-symptomatic individuals and elevated in AD patients [11]. Our study complements the findings from Wingo et al. [12], which observed that elevated apoB was significantly associated with increased risk of early onset AD (EOAD) and EOAD cases were enriched for apoB rare functional variants (Supplementary Note, Additional File 1). However, mendelian randomization (MR) analysis assessing the causal effect of statin-adjusted apoB [13] on AD risk [14] were inconclusive due to confounding by the *APOE* region, and significant evidence of heterogeneity and horizontal pleiotropy (with and without the APOE region: Supplementary Table 9, Additional File 1), which violated the assumptions underlying this methodology. MR analysis of apoB *cis* protein quantitative trait loci [15] and AD risk [14] were not significant. Colocalization analysis does not suggest evidence of a shared causal variant for apoB and AD. Follow-up mechanistic studies are critical to establish the relationship between APOECh, apoB levels, and AD risk.

This study includes several limitations. We did not identify any homozygotes for APOECh and are unable to validate the findings reported by Arboleda-Velasquez et al. [5]. Due to the rarity of the APOECh variant, the sample size of carriers is insufficient for leveraging UKB brain imaging, metabolomics, and proteomics data, which are currently only available on partially overlapping subsets of UKB participants; the overlap with carriers was too small to perform statistical analyses (Supplementary Table 10, Additional File 1). For the quantitative traits assessed, only baseline measurements were considered given that repeated biomarker measurements are available only for a subset of UKB participants. Statin adjustment for lipid biomarkers were only applied using self-reported statin at baseline. Primary care prescription data were not considered for statin adjustment because only 48.8% of the UKB cohort has prescription data (Supplementary Table 11, Additional File 1). Statin adjustment may be incomplete because not all individuals report medication use; confounders assessed by self-report may be sub-optimally controlled depending on the outcome of interest [16]. Larger population-based cohorts such as All of US (N ~ 1 million) or Our Future Health (N ~ 5 million) will enable replication studies to confirm whether APOECh carriers have lower apoB levels. Assessment of linkage disequilibrium and/or an interaction effect of the APOECh variant with other AD-protective variants to establish the mechanism by which heterozygous carriers may have a protective effect is an important next step.

To our knowledge, this work is the first examination of the clinical phenotypes in the largest cohort of APOECh carriers among UKB participants. While the APOECh variant is very rare and larger cohorts are needed to assess its contribution to dementia, dyslipidemia, and CVD, the UKB provides an unprecedented opportunity to follow these carriers and elucidate the underlying role of APOECh in disease etiology.

### Supplementary Information

**Additional file 1:** **Table S1.** Characteristics of APOE Christchurch variant carriers and matched noncarriers in UKB European samples. **Table S2.** List of self-reported cardiovascular conditions included for analyses. **Table S3.** List of self-reported and primary care prescription medications used for analyses. **Table S4.** List of quantitative traits assessed for carriers and noncarriers. **Table S5.** *APOE* genotype of APOECh carriers and matched noncarriers. **Table S6.** Screening APOECh carriers for APOE protective variants and mutations reported for neurodegenerative disorders with a Mendelian inheritance. **Table S7.** Empirical p-values of binary traits. **Table S8.** Kolmogorov-Smirnov test of quantitative traits and PRS among all 36 carriers and matching controls. **Table S9.** Mendelian randomization of statin-adjusted apoB and AD. **Table S10.** Availability and overlap of APOECh carriers with brain imaging, metabolomics, and proteomics data. **Table S11.** Summary of phenotypic data availability for carriers and noncarriers. **Table S12.** Linear and logistic regression of HDL, LDL, CVD, and AD with PRS as a covariate between APOECh carriers and noncarriers. **Figure S1.** Selection of APOE Christchurch carriers and matched controls from the UK Biobank. **Figure S2.** Cumulative distributions of physical measures and Kolmogorov-Smirnov test results. **Figure S3.** Cumulative distributions of urine biomarkers and Kolmogorov-Smirnov test results. **Figure S4.** Cumulative distributions of blood biomarkers and Kolmogorov-Smirnov test results. **Figure S5.** Cumulative distributions of hematological traits and Kolmogorov-Smirnov test results. **Figure S6.** Cumulative distributions of lipid biomarkers and Kolmogorov-Smirnov test results.

## Data Availability

The full set of analytical results is available within the manuscript and/or online supplement. Data used in the preparation of this article were obtained from the UK Biobank (https://www.ukbiobank.ac.uk/researchers/). All *bona fide* researchers from academic, commercial, and charitable organizations can apply access to the UK Biobank resource to conduct health-related research that is in the public interest.

## Abbreviations

AD: Alzheimer’s disease
apoB: Apolipoprotein B
apoE: Apolipoprotein E
APOECh: APOE Christchurch
CVD: Cardiovascular disease
MACE: Major adverse cardiovascular event
PRS: Polygenic risk score
UKB: UK Biobank
WES: Whole exome sequencing
WGS: Whole genome sequencing
