# Supplementary material for: Characterization of APOE Christchurch carriers in 455,306 UK Biobank participants
Source: Mol Neurodegener. 2023 Nov 28;18:92. doi: 10.1186/s13024-023-00684-7 (PMC10685495; doi:10.1186/s13024-023-00684-7)
Supplement: Supplementary file 1 — Additional file 1: Table S1. Characteristics of APOE Christchurch variant carriers and matched noncarriers in UKB European samples. Table S2. List of self-reported cardiovascular conditions included for analyses. Table S3. List of self-reported and primary care prescription medications used for analyses. Table S4. List of quantitative traits assessed for carriers and noncarriers. Table S5. APOE genotype of APOECh carriers and matched noncarriers. Table S6. Screening APOECh carriers for APOE protective variants and mutations reported for neurodegenerative disorders with a Mendelian inheritance. Table S7. Empirical p-values of binary traits. Table S8. Kolmogorov-Smirnov test of quantitative traits and PRS among all 36 carriers and matching controls. Table S9. Mendelian randomization of statin-adjusted apoB and AD. Table S10. Availability and overlap of APOECh carriers with brain imaging, metabolomics, and proteomics data. Table S11. Summary of phenotypic data availability for carriers and noncarriers. Table S12. Linear and logistic regression of HDL, LDL, CVD, and AD with PRS as a covariate between APOECh carriers and noncarriers. Figure S1. Selection of APOE Christchurch carriers and matched controls from the UK Biobank. Figure S2. Cumulative distributions of physical measures and Kolmogorov-Smirnov test results. Figure S3. Cumulative distributions of urine biomarkers and Kolmogorov-Smirnov test results. Figure S4. Cumulative distributions of blood biomarkers and Kolmogorov-Smirnov test results. Figure S5. Cumulative distributions of hematological traits and Kolmogorov-Smirnov test results. Figure S6. Cumulative distributions of lipid biomarkers and Kolmogorov-Smirnov test results. [file 13024_2023_684_MOESM1_ESM.docx]

**Supplementary Material**

**Supplementary Methods.** Additional information on sample sequencing, phenotype definitions, and statistical analyses

**Supplementary Note.**

Descriptions of deceased APOECh carriers

Comparisons of polygenic risk scores in carriers and noncarriers

Links between apoB and AD in the literature

**Supplementary Table 1.** Characteristics of APOE Christchurch variant carriers and matched noncarriers in UKB European samples

**Supplementary Table 2.** List of self-reported cardiovascular conditions included for analyses

**Supplementary Table 3.** List of self-reported and primary care prescription medications used for analyses

**Supplementary Table 4.** List of quantitative traits assessed for carriers and noncarriers

**Supplementary Table 5.** *APOE* genotype of APOECh carriers and matched noncarriers

**Supplementary Table 6.** Screening APOECh carriers for APOE protective variants and mutations reported for neurodegenerative disorders with a Mendelian inheritance

**Supplementary Table 7.** Empirical p-values of binary traits

**Supplementary Table 8.** Kolmogorov-Smirnov test of quantitative traits and PRS among all 36 carriers and matching controls

**Supplementary Table 9.** Mendelian randomization of statin-adjusted apoB and AD

**Supplementary Table 10.** Availability and overlap of APOECh carriers with brain imaging, metabolomics, and proteomics data

**Supplementary Table 11.** Summary of phenotypic data availability for carriers and noncarriers

**Supplementary Table 12.** Linear and logistic regression of HDL, LDL, CVD, and AD with PRS as a covariate between APOECh carriers and noncarriers

**Supplementary Figure 1.** Selection of APOE Christchurch carriers and matched controls from the UK Biobank

**Supplementary Figure 2.** Cumulative distributions of physical measures and Kolmogorov-Smirnov test results

**Supplementary Figure 3.** Cumulative distributions of urine biomarkers and Kolmogorov-Smirnov test results

**Supplementary Figure 4.** Cumulative distributions of blood biomarkers and Kolmogorov-Smirnov test results

**Supplementary Figure 5.** Cumulative distributions of hematological traits and Kolmogorov-Smirnov test results

**Supplementary Figure 6.** Cumulative distributions of lipid biomarkers and Kolmogorov-Smirnov test results

**Supplementary References.** References in Supplementary Methods, Tables, and Figures

**Supplementary Methods**

***Participants***

The UKB is a prospective cohort study with deep genetic and rich phenotypic data on nearly half a million individuals (1). At the time this study was completed, WES and WGS data were available for ~450,000 (2) and ~150,000 individuals, respectively (3). The UKB study was conducted under generic approval from the NHS National Research Ethics Service (approval letter dated 17th June 2011, Ref 11/NW/0382). All participants gave full informed written consent. This research has been conducted using the UK Biobank—project number 52293. Phenotypic data were derived based on the ukb50945 March 2022 data release, while education years (EDU_YR), fluid intelligence score (FIS), and reaction time (RT) were extracted from ukb49194 October 2021.

***Sequencing***

WES data for UK Biobank participants were generated at the Regeneron Genetics Center (RGC) as part of a public-private partnership between eight pharmaceutical companies (AbbVie, Alnylam Pharmaceuticals, AstraZeneca, Biogen, Bristol-Myers Squibb, Pfizer, Regeneron, and Takeda) and UK Biobank. Briefly, genomic DNA underwent paired-end 75bp WES at RGC using the IDT xGen v1 capture kit on NovaSeq6000 machines. Initial QC was performed by RGC and included checks for sex discordance, contamination, unresolved duplicate sequences, and discordance with microarray genotyping data. Detailed methods for joint variant calling, QC thresholds, and filtering criteria have been previously described by UK Biobank Exome Sequencing Consoritium (UKB-ESC) (4). WES data from 450,000 UK Biobank participants were made publicly available in October 2021 and underwent internal QC and filtration in a manner similar to that of UKB-ESC prior to ingestion and analysis.

WGS data for UK Biobank participants were generated from sequencing efforts at the Wellcome Sanger Institute and deCODE as part of a public-private partnership involving Amgen, AstraZeneca, GlaxoSmithKline, and Janssen Pharmaceutical Companies of Johnson & Johnson, alongside Wellcome and UK Research and Innovation (UKRI). Briefly, 150 bp paired-end sequencing was performed on an Illumina NovaSeq 6000. Per-sample quality checks were performed (e.g., yield, read count, GC fraction, insert fragment size distribution, etc.), with samples passing checks proceeding to genotyping. Data were aligned to the GRCh38 reference genome. Samples were checked for contamination, sex mismatch, and concordance with microarray-called genotypes. Joint-genotyped variants were called using the Genome Analysis Toolkit (GATK) and Graphtyper; we focus on the GATK-called dataset here. Data underwent a multistep QC process. For autosomes, hemizygous genotypes were masked, as were any genotypes with genotype qualities (GQ) less than 30. Indel genotype calls were masked if they had a depth (DP) less than 10; SNP calls were masked if they had a DP less than 20. Additionally, variants (sites) were subjected to a series of filters following the Broad Institute’s recommendations for hard filtering (SNPs: QD < 2, QUAL < 30, SOR > 3, FS > 60, MQ < 40, MQRankSum < -12.5, ReadPosRankSum < -8; indels: as before, but with FS > 200 and ReadPosRankSum < -20; per-site missingness > 10%). The same filters were applied for the X chromosome, but variant genotypes were masked if they had a DP less than 10. WGS data from 141,948 UK Biobank participants were made available to us in July 2021 after internal QC (including removal of duplicated variants).

***Genotype screening***

*APOE* genotype (**Supplementary Table 5**), *APOE* protective variants, and mutations for neurodegenerative disorders with evidence of Mendelian inheritance in *APP*, *PSEN1*, *PSEN2*, *GRN*, *SORT1*, *MAPT,* and *APBB2* were screened for both carriers and noncarriers of APOECh using WES and WGS data (**Supplementary Table 6**). In *APOE*, we examined rs429358-C, rs7412-T, and the missense APOECh variant rs121918393-A. We also examined whether any of the APOECh carriers had V236E Jacksonville (rs199768005-A) and R251G (rs267606661-G) protective alleles.

***Phenotype definitions***

The phenotype data collected includes self-reported information, standardized assessments, hospital and primary care records, cancer and death registries, quantitative biomarkers, infectious disease antigens, and multi-modal imaging results for up to 500,000 individuals. Birth date was derived using year and month of birth (UKB Data Field 34 and 52). Current age is based on the individuals’ age as of January 1, 2022; age-at-death was assessed for deceased subjects using UKB Data Field 40007.

Alzheimer's Disease (AD) is defined as ICD-10 codes F00, F00.1, F00.2, F00.9, G30, G30.0, G30.1, G30.8, and/or G30.9 in UKB Data Fields 41270 (ICD-10 diagnoses), 40001 (ICD-10 primary cause of death), and 40002 (ICD-10 secondary cause of death), ICD-9 code 331 in UKB Data Field 41271 (ICD-9 diagnoses), and self-reported code 1263 in UKB Data Field 20002 (self-reported non-cancer illness). Family history (FH) of AD was ascertained using self-reported code 10 from UKB Data Fields 20110 and 20107 (illnesses of mother and father, respectively); the subject is characterized as having FH if at least 1 parent was reported to have AD. MCI and other cognitive function symptoms were defined using ICD-10 codes F06.7, R41, and R41.8 and ICD-9 code 3101 in UKB Data Fields 41270, 40001 40002, and 41271. CVD was broadly defined as any ICD-10 codes in chapter 9 I00-I99 (diseases of the circulatory system), chapter 18 R00-R09 (symptoms and signs involving circulatory and respiratory systems), or chapter 21 Z867 (personal history of disease of the circulatory system), ICD-9 codes in chapter 7 (390-459), chapter 16 785-786 (symptoms involving cardiovascular system, respiratory system, and other chest symptoms), and self-reported history of any cardiovascular conditions (UKB Data Field 20002; **Supplementary Table 2**). Dyslipidemia was defined as ICD-10 codes E78 and E78.0-E78.9, ICD-9 codes 2720-2729, and self-reported code 1473 for high cholesterol (UKB Data Field 20002). Use of lipid-lowering drugs include primary care prescription data from any time point under ATC codes beginning with prefix C10 and any self-reported use of statins and other types of non-statin lipid-lowering drugs (UKB Data Field 20003; **Supplementary Table 3**). Hypertension was defined as ICD-10 code I10 (essential hypertension) and ICD-9 codes for 401, 4010, 4011, and 4019 (essential hypertension). Use of antihypertensive include primary care prescription data from any time point under ATC codes beginning with prefixes C02, C03, C07, C08, and C09 and any self-reported use of antihypertensive drugs (UKB Data Field 20003; **Supplementary Table 3**). Type 2 diabetes was defined as ICD-10 codes E11, ICD-9 codes 250.*0 and 250.*2, and self-reported code 1223 for type 2 diabetes (UKB Data Field 42000). Major adverse cardiovascular event (MACE) was defined as ICD-10 codes I21-I25, ICD-9 codes 410, 412, 414, self-reported code 1075 for heart attack/myocardial infarction (UKB Data Field 20002), operative procedures OPCS4 codes K40-K46, K49-K50, K75, and any record of myocardial infarction outcomes (UKB Data Field 42000). Cerebral amyloid angiopathy was defined as ICD-10 code I68.0 and ICD-9 code 277.39. Vascular dementia was defined as ICD-10 codes F01.0-F01.3 and F01.8-F01.9 and ICD-9 code 290.40. Parkinson’s disease was defined as ICD-10 codes G20, ICD-9 codes 332, and self-reported code 1262 for Parkinson’s disease (UKB Data Field 20002). Depression was defined as ICD-10 codes F20.4, F32.8, and F33.0-F33.9, ICD-9 codes 296.2, 296.22-296.26, 296.3, 296.32-296.36, and 311, and self-reported code 1286 for depression (UKB Data Field 20002). Schizophrenia was defined as ICD-10 codes F20, F20.0-F20.9, F25, F25.0-F25.9, ICD-9 codes 295.0-295.9, and self-reported code 1289 for schizophrenia (UKB Data Field 20002). Bipolar disorder was defined as ICD-10 codes F30.0, F30.1, F30.8, F30.9, F31.0-F31.9, ICD-9 codes 296.0, 296.1, 296.4-296.8, and self-reported code 1291 for bipolar disorder (UKB Data Field 20002).

A total of 80 quantitative traits were assessed (**Supplementary Table 4**). Blood and urine biomarkers, hematologic traits, physical measures, education years, and cognitive traits (fluid intelligence score, and reaction time) were ascertained at assessment centers and the first instance at baseline were used for evaluation. LDL, cholesterol, and apoB have been adjusted for self-reported statin at baseline as described in the study published by Sinnott-Armstrong and Tanigawa (5). Systolic and diastolic blood pressure have been adjusted for self-reported antihypertensive at baseline (6). Primary care prescription use of other types of lipid-lowering drugs and antihypertensive were not considered for medication adjustment given primary care is only available for approximately half of the entire UKB cohort. Quantitative traits with multiple measurements from the same visit (e.g., blood pressure) were averaged before comparing carriers to noncarriers. Education attainment was converted to education years based on UKB Data Fields 6138 (educational qualifications) and 10722 (education qualifications pilot); the maximum value is considered as the highest education completed (7). Additionally, standard polygenic risk scores (8) for 2 lipid traits (high-density lipoprotein [HDL] and low-density lipoprotein), 5 cardiovascular events (atrial fibrillation, cardiovascular disease, coronary artery disease, hypertension, and ischaemic stroke), and 3 neurological diseases (Alzheimer’s disease, multiple sclerosis, and Parkinson’s disease) were extracted from ukb669060 (ascertained August 2022) and compared to assess whether genetic risk differs between carriers and noncarriers.

***Medical records data availability***

Phenotypic data availability varies across individuals in the UKB. Thirty out of thirty-seven APOECh carriers have at least 1 ICD-9 (UKB Data Field 41271) or ICD-10 code (UKB Data Field 41270), 24 have at least one self-reported non-cancer illness at the time of baseline assessment (UKB Data Field 20002), 30 have at least one self-reported medication at the time of baseline assessment (UKB Data Field 20003), 18 have at least one primary care record and primary care prescription record (gp_scripts), and 32 have at least 1 lipid biomarker measurement at baseline (UKB Data Fields 30630, 30640, 30710, 30690, 30760, 30780, 30790, 30870). The proportion of data available for each category is shown in **Supplementary Table 11**.

***Study design and statistical analysis***

In this study (**Supplementary Figure 1**), we compared the clinical profiles of 36 European APOECh carriers and 129,240 matched noncarriers for 19 binary traits, 80 quantitative traits, and 10 polygenic risk scores (PRS) described in Phenotype Definitions. To avoid confounding by genetic ancestry and due to a lack of available clinical information, the only APOECh carrier with admixed American ancestry was excluded from downstream statistical analyses. We identified a noncarrier cohort by maximizing the number of matching sets that could be generated from among UKB participants given age (5-year bin), genetic sex, and genetic ancestry from Pan-UKBB (UKB Return Field 31063) (9) resulting in a 1:3,590 ratio (36 carriers:129,240 noncarriers).

Medical history, including ICD-10 diagnoses (UKB Data Field 41270), ICD-9 diagnoses (UKB Data Field 41271), ICD-10 primary cause of death (UKB Data Field 40001), ICD-10 secondary cause of death (UKB Data Field 40002), self-reported non-cancer illness (UKB Data Field 20002) and medications (UKB Data Field 20003), as well as primary care data and prescriptions (gp_scripts) ascertained from baseline assessment center visit were evaluated to determine whether specific medical characteristics are enriched in the APOECh carriers (**Supplementary Methods: Phenotype definitions; Supplementary Tables 2 and 3**). Only 9 carriers had metabolomics, 7 had proteomics, and 3 had brain imaging data (**Supplementary Table 10**). Given only 37 carriers, of which 36 Europeans are retained for analysis, we focused on assessing the difference between carriers and noncarriers in medical history for AD and related cognitive traits, cardiovascular health, and quantitative traits. Additionally, we explored whether PRS differed between carriers and noncarriers to determine whether their genetic background for developing cardiovascular and neurodegenerative diseases could differentially influence disease risk.

For binary traits assessed in this study, the cumulative probability function was calculated using R to assess whether carrier event frequency is lower or higher than expected based on noncarrier prevalence. For quantitative traits, a two-sided Kolmogorov-Smirnov (KS) test in R (ks.test) was used to test whether the distributions for carriers vs. noncarriers differed significantly. Baseline values measured at enrollment were used for quantitative traits as they have the greatest number of observations. Longitudinal effects were not assessed due to missingness and loss to follow-up.

***Sensitivity analysis of medication usage in individuals with prescription data***

Given primary care prescription data is only available for 48.8% of the UKB cohort, we performed a sensitivity analysis to assess whether there is a difference in medication use among carriers and noncarriers with prescription data. We further subsetted the study sample cohort to 17 European APOECh carriers and 27,013 matched noncarriers with prescription data. The 27,013 noncarriers included for assessment were extracted from the pool of 129,240 noncarriers that were originally matched based on sex, age, and ancestry presented as the primary analysis of this study. We compared the frequencies of lipid-lowering and antihypertensive drugs in carriers and non-carriers using binomial distribution probability.

***Mendelian randomization of statin-adjusted apoB and AD***

To assess whether there is a causal effect between statin-adjusted apoB and AD risk, we performed a Mendelian randomization (MR) analysis with external summary statistics using the *TwoSampleMR* R package. To generate the genetic instruments, we subsetted SNPs from statin-adjusted apoB GWAS (5) reaching genome-wide significance threshold (p < 5 x 10^-8^) and further completed LD clumping (R^2^ < 0.1, window size of 500kb), resulting in over 900 SNPs as instrumental variables. Summary statistics for the outcome were extracted from the most recent large-scale AD GWAS meta-analysis to date (10). Four MR approaches were assessed, including MR-Egger, inverse variance weighted, weighted mode, and weighted median (**Supplementary Table 9**). Given several GWAS signals for statin-adjusted apoB come from the *APOE* gene region, all MR analyses were repeated after the exclusion of the APOE SNPs (hg38: chr19:44905796-44909393). We evaluated causal effect heterogeneity and horizontal pleiotropy as sensitivity analyses.

Additionally, to avoid the confounding effect of *APOE* and other unknown factors, we performed MR analysis with *cis* protein quantitative trait loci (*cis*-pQTLs) of apoB. Given there are no significant *cis*-pQTLs of apoB in the UKB Pharma Proteomic Project, we used the 4 cis-pQTLs from a deCODE study (11) as instrumental variables and the Bellenguez et al. (10) AD GWAS study for the outcome. We carried out the MR analyses using the same approaches listed above.

***Colocalization of statin-adjusted apoB and AD***

To assess whether apoB and AD share the same causal variant, we performed colocalization analysis with the *coloc* R package with the same statin-adjusted apoB (5) and AD (10) GWAS summary statistics used in the MR analysis. We defined the *APOB* region based on the NCBI gene region (hg38: chr2 21001429-21044073) +/- 500kb flanking region. Then we assessed posterior probability H4 to determine whether the two traits share a causal variant.

**Supplementary Note**

***Descriptions of deceased APOECh carriers***

Given the important role of *APOE* and the hypothesized role of APOECh in dyslipidemias and CVD risk, we examined the cardiovascular and metabolic health of APOECh carriers. One participant died at the age of 72 years from acute myocardial infarction (primary cause of death, UKB Data Field 40001) and atherosclerotic heart disease of native coronary artery (secondary cause of death, UKB Data Field 40002). Another participant died at the age of 67 from hypertensive heart disease (primary cause of death, UKB Data Field 40001). However, there is no difference in occurrence of CVD (p=0.34), dyslipidemia (p=0.13) and hypertension (p=0.24) as defined based on ICD codes and self-reported data when comparing all carriers to noncarriers.

***Comparisons of polygenic risk scores in carriers and noncarriers***

Our analyses demonstrated that the polygenic risk scores for HDL, LDL, and polygenic risk for atrial fibrillation, cardiovascular disease, coronary artery disease, hypertension, and stroke do not differ between carriers and noncarriers (**Supplementary Table 8**). Furthermore, linear and logistic regression analyses with polygenic score (HDL and LDL) or polygenic risk score (CVD, hypertension, and AD) as a covariate for quantitative and binary traits, respectively, showed that there is no difference in HDL/LDL levels, CVD, and AD between carriers and noncarriers (**Supplementary Table 12**).

***Links between apoB and AD in the literature***

Our study complements the findings from Wingo et al. 2019 (12), which observed that elevated apoB was significantly associated with increased risk of EOAD but was only partially mediated by APOE4. However, Wingo et al. did not adjust for other *APOE* genotypes beyond APOE4, such as those from APOE3Ch. Additionally, the variants included in their *APOB* rare variant analysis do not have robust functional evidence for either LDL levels or LOAD, suggesting that there are other factors linked contributing to apoB levels and AD risk. Therefore, findings from Wingo et al. and this study both highlight the importance of cholesterol metabolism in AD pathogenesis and further studies are needed to fully understand the mechanistic link between apoB levels and AD risk.

CSF proteomic data is not available for the UK Biobank; therefore, no comparisons can be made between plasma and CSF apoB levels for the study participants. No significant correlations have been found in plasma and CSF apoB (13). ApoB is not typically found in the CNS and plasma apoB have been found to positively correlate with A-beta plaque abundance in AD transgenic mice models (14), suggesting a compromised blood brain barrier may contribute to AD pathology (15). Overexpression of apoB in the serum of transgenic mice have shown to alter the cerebral protein profile and trigger apoptosis and neurodegeneration in the brain (16).

**Supplementary Tables**

Supplementary Table 1. Characteristics of *APOE* Christchurch variant carriers and matched noncarriers in UKB European samples

| ​ | Carriers (N=37)​ | European Noncarriers (N=129,240) |
| --- | --- | --- |
| Age | Median: 68.63  Mean: 68.91  Min: 56.62  Max: 82.06 | Median: 68.38  Mean: 68.86  Min: 56  Max: 82.97 |
| Genetic sex​ | 27 females​  10 males​ | 93,340 females  35,900 males |
| Pan-UKBB derived ancestry​ | 36 Europeans  1 admixed American | 129,240 Europeans |
| Deceased participants | 2 deceased  Primary causes of death: acute myocardial infarction (MI), hypertensive heart disease without heart failure | 8,779 deceased  Primary causes of death: lung cancer (9.4%), breast cancer (5.8%), pancreatic cancer (3.8%), acute MI (3.3%), chronic ischaemic heart disease (2.7%) |
| Alzheimer’s disease (ICD + self-report)​ | None​ | 660 (0.51%)  643 ICD code; 39 self-reported |
| Parental history of Alzheimer’s disease​ | 4 (10.8%) with at least 1 parent (2 paternal; 2 maternal) | 17,428 (13.5%) with at least 1 parent (6500 paternal; 11705 maternal) |
| Mild cognitive impairment or other cognitive symptoms ICD codes | None​ | 755 (0.58%) |

Demographic and partial disease profiling of APOECh carriers and matched noncarriers. Noncarriers were matched by ancestry, sex, and 5-year age bin to 36 European APOECh carriers with 1:3590 ratio.

Supplementary Table 2. List of self-reported cardiovascular conditions included for analyses

| Data Field/Table | Category | Code | Description |
| --- | --- | --- | --- |
| 20002 | Self-reported cardiovascular conditions | 1065 | hypertension |
|  |  | 1066 | heart/cardiac problem |
|  |  | 1072 | essential hypertension |
|  |  | 1074 | angina |
|  |  | 1075 | heart attack/myocardial infarction |
|  |  | 1076 | heart failure/pulmonary odema |
|  |  | 1077 | heart arrhythmia |
|  |  | 1471 | atrial fibrillation |
|  |  | 1483 | atrial flutter |
|  |  | 1484 | wolff parkinson white / wpw syndrome |
|  |  | 1485 | irregular heart beat |
|  |  | 1486 | sick sinus syndrome |
|  |  | 1487 | svt / supraventricular tachycardia |
|  |  | 1078 | heart valve problem/heart murmur |
|  |  | 1584 | mitral valve disease |
|  |  | 1488 | mitral valve prolapse |
|  |  | 1489 | mitral stenosis |
|  |  | 1585 | mitral regurgitation / incompetence |
|  |  | 1586 | aortic valve disease |
|  |  | 1490 | aortic stenosis |
|  |  | 1587 | aortic regurgitation / incompetence |
|  |  | 1079 | cardiomyopathy |
|  |  | 1588 | hypertrophic cardiomyopathy (hcm / hocm) |
|  |  | 1080 | pericardial problem |
|  |  | 1589 | pericarditis |
|  |  | 1590 | pericardial effusion |
|  |  | 1426 | myocarditis |
|  |  | 1479 | rheumatic fever |
|  |  | 1081 | stroke |
|  |  | 1086 | subarachnoid haemorrhage |
|  |  | 1491 | brain haemorrhage |
|  |  | 1583 | ischaemic stroke |
|  |  | 1082 | transient ischaemic attack (tia) |
|  |  | 1083 | subdural haemorrhage/haematoma |
|  |  | 1425 | cerebral aneurysm |
|  |  | 1067 | peripheral vascular disease |
|  |  | 1087 | leg claudication/ intermittent claudication |
|  |  | 1088 | arterial embolism |
|  |  | 1492 | aortic aneurysm |
|  |  | 1591 | aortic aneurysm rupture |
|  |  | 1592 | aortic dissection |
|  |  | 1068 | venous thromboembolic disease |
|  |  | 1093 | pulmonary embolism +/- dvt |
|  |  | 1094 | deep venous thrombosis (dvt) |
|  |  | 1473 | high cholesterol |
|  |  | 1493 | other venous/lymphatic disease |
|  |  | 1494 | varicose veins |
|  |  | 1495 | lymphoedema |
|  |  | 1593 | varicose ulcer |

Data field 20002 (non-cancer illness code, self-reported) and data codes correspond to UK Biobank main tabular phenotype data. More details can be found on the UKB Showcase ([Data Field 20002](https://biobank.ndph.ox.ac.uk/ukb/field.cgi?id=20002)).

Supplementary Table 3. List of self-reported and primary care prescription medications used for analyses

| Data Field/Table | Category | Code | Description |
| --- | --- | --- | --- |
| 20003 | Self-reported statins | 1140861958 | simvastatin |
|  |  | 1140869130 | ecostatin 150mg pessary |
|  |  | 1140869132 | ecostatin twinpack |
|  |  | 1140869196 | ecostatin-1 150mg pessary |
|  |  | 1140870208 | sandostatin 50micrograms/1ml injection |
|  |  | 1140873350 | imipenem + cilastatin |
|  |  | 1140873570 | tetracycline+nystatin 250mg/250ku tablet |
|  |  | 1140874030 | metronidazole+nystatin 400mg/10000units tablet+pessary |
|  |  | 1140874266 | nystatin dome 100,000units/ml oral suspension |
|  |  | 1140874360 | nystatin |
|  |  | 1140878594 | ecostatin cream |
|  |  | 1140878598 | ecostatin lotion |
|  |  | 1140880388 | nystatin+tolnaftate 100000units/1%/g cream |
|  |  | 1140880390 | nystatin+chlorhexidine hydrochloride 100000units/1%/g cream |
|  |  | 1140882794 | clobetasol propionate+neomycin sulphate+nystatin |
|  |  | 1140882806 | clobetasone butyrate+oxytetracycline+nystatin |
|  |  | 1140882844 | hydrocortisone+nystatin |
|  |  | 1140882938 | terra-cortril nystatin cream |
|  |  | 1140883060 | triamcinolone+nystatin |
|  |  | 1140884216 | ecostatin 1% powder |
|  |  | 1140888594 | fluvastatin |
|  |  | 1140888648 | pravastatin |
|  |  | 1140910632 | eptastatin |
|  |  | 1140910654 | velastatin |
|  |  | 1141146234 | atorvastatin |
|  |  | 1141157400 | nystatin product |
|  |  | 1141192410 | rosuvastatin |
| 20003 | Self-reported non-statin lipid lowering medications | 1140861924 | bezafibrate |
|  |  | 1140861942 | cholestyramine+aspartame 4g/sachet powder |
|  |  | 1140861944 | clofibrate |
|  |  | 1140861954 | fenofibrate |
|  |  | 1140862026 | ciprofibrate |
|  |  | 1140865576 | cholestyramine |
|  |  | 1140888590 | colestipol |
|  |  | 1140910670 | niacin |
|  |  | 1141157260 | bezafibrate product |
|  |  | 1141157262 | gemfibrozil product |
|  |  | 1141157416 | cholestyramine product |
|  |  | 1141192736 | ezetimibe |
| 20003 | Self-reported anti-hypertensive | 1140860192 | nadolol |
|  |  | 1140860292 | pindolol |
|  |  | 1140860308 | metoprolol tartrate+chlorthalidone 100mg/12.5mg tablet |
|  |  | 1140860312 | nadolol+bendrofluazide 40mg/5mg tablet |
|  |  | 1140860316 | nadolol+bendrofluazide 80mg/5mg tablet |
|  |  | 1140860322 | pindolol+clopamide 10mg/5mg tablet |
|  |  | 1140860332 | sotalol hydrochloride+hydrochlorothiazide 80mg/12.5mg tablet |
|  |  | 1140860336 | timolol maleate+co-amilozide 10mg/2.5mg/25mg tablet |
|  |  | 1140860340 | timolol maleate+bendrofluazide 10mg/2.5mg tablet |
|  |  | 1140860342 | timolol maleate+bendrofluazide 20mg/5mg tablet |
|  |  | 1140860404 | metoprolol tartrate+hydrochlorothiazide 100mg/12.5mg tablet |
|  |  | 1140860418 | propranolol hydrochloride+bendrofluazide 80mg/2.5mg capsule |
|  |  | 1140860422 | acebutolol+hydrochlorothiazide 200mg/12.5mg tablet |
|  |  | 1140860426 | atenolol+nifedipine 50mg/20mg m/r capsule |
|  |  | 1140860470 | methyldopa |
|  |  | 1140860532 | minoxidil |
|  |  | 1140860562 | methyldopa+hydrochlorothiazide 250mg/15mg tablet |
|  |  | 1140860696 | lisinopril |
|  |  | 1140860728 | quinapril |
|  |  | 1140860738 | quinalapril+hydrochlorothiazide 10mg/12.5mg tablet |
|  |  | 1140860750 | captopril |
|  |  | 1140860764 | captopril+hydrochlorothiazide 25mg/12.5mg tablet |
|  |  | 1140860790 | enalapril maleate+hydrochlorothiazide 20mg/12.5mg tablet |
|  |  | 1140860806 | ramipril |
|  |  | 1140860904 | trandolapril |
|  |  | 1140861088 | nifedipine |
|  |  | 1140861190 | isradipine |
|  |  | 1140864202 | chlorthalidone tablet+potassium m/r tablet 25mg/6.7mmol pack |
|  |  | 1140864950 | bisoprolol fumarate+hydrochlorothiazide 10mg/6.25mg tablet |
|  |  | 1140864952 | lisinopril+hydrochlorothiazide 10mg/12.5mg tablet |
|  |  | 1140866078 | indapamide |
|  |  | 1140866092 | metolazone |
|  |  | 1140866138 | chlorothiazide |
|  |  | 1140866144 | chlorthalidone |
|  |  | 1140866162 | hydrochlorothiazide |
|  |  | 1140866236 | spironolactone |
|  |  | 1140866280 | bumetanide |
|  |  | 1140866324 | triamterene+benzthiazide 50mg/25mg capsule |
|  |  | 1140866330 | triamterene+chlorthalidone 50mg/50mg tablet |
|  |  | 1140866332 | triamterene+frusemide 50mg/40mg tablet |
|  |  | 1140866388 | triamterene |
|  |  | 1140866422 | amiloride hcl+cyclopenthiazide 2.5mg/250micrograms tablet |
|  |  | 1140866426 | amiloride hydrochloride+bumetanide 5mg/1mg tablet |
|  |  | 1140866448 | bumetanide+potassium 500micrograms/7.7mmol m/r tablet |
|  |  | 1140866724 | acebutolol |
|  |  | 1140866738 | atenolol |
|  |  | 1140871986 | clonidine hydrochloride 25micrograms tablet |
|  |  | 1140875840 | timolol 0.25% eye drops |
|  |  | 1140875934 | apraclonidine |
|  |  | 1140879758 | betaxolol |
|  |  | 1140879760 | bisoprolol |
|  |  | 1140879778 | doxazosin |
|  |  | 1140879794 | prazosin |
|  |  | 1140879798 | terazosin |
|  |  | 1140879802 | amlodipine |
|  |  | 1140879806 | diltiazem |
|  |  | 1140879810 | nicardipine |
|  |  | 1140879824 | labetalol |
|  |  | 1140879842 | propranolol |
|  |  | 1140879866 | timolol |
|  |  | 1140883468 | clonidine |
|  |  | 1140888510 | verapamil |
|  |  | 1140888512 | amiloride |
|  |  | 1140888552 | enalapril |
|  |  | 1140888556 | fosinopril |
|  |  | 1140888560 | perindopril |
|  |  | 1140888646 | felodipine |
|  |  | 1140888686 | hydralazine |
|  |  | 1140909368 | carvedilol |
|  |  | 1140909708 | furosemide |
|  |  | 1140910606 | alpha methyldopa |
|  |  | 1140916356 | losartan |
|  |  | 1140923712 | moexipril |
|  |  | 1140926778 | diltiazem hcl+hydrochlorothiazide 150mg/12.5mg m/r capsule |
|  |  | 1140928226 | nisoldipine |
|  |  | 1141145660 | valsartan |
|  |  | 1141146124 | atenolol+chlorthalidone |
|  |  | 1141146126 | atenolol+bendrofluazide |
|  |  | 1141146128 | atenolol+co-amilozide |
|  |  | 1141151016 | losartan potassium+hydrochlorothiazide 50mg/12.5mg tablet |
|  |  | 1141152998 | irbesartan |
|  |  | 1141153328 | trandolapril+verapamil hydrochloride |
|  |  | 1141156836 | candesartan cilexetil |
|  |  | 1141165470 | felodipine+ramipril |
|  |  | 1141166006 | telmisartan |
|  |  | 1141169516 | dorzolamide+timolol |
|  |  | 1141171336 | eprosartan |
|  |  | 1141172682 | irbesartan+hydrochlorothiazide 150mg/12.5mg tablet |
|  |  | 1141180592 | perindopril+indapamide |
|  |  | 1141180772 | triamterene+chlortalidone 50mg/50mg tablet |
|  |  | 1141180778 | atenolol+chlortalidone |
|  |  | 1141184722 | latanoprost+timolol |
|  |  | 1141187788 | telmisartan+hydrochlorothiazide 40mg/12.5mg tablet |
|  |  | 1141194804 | nadolol+endroflumethiazide 40mg/5mg tablet |
|  |  | 1141194808 | timolol maleate+endroflumethiazide 10mg/2.5mg tablet |
|  |  | 1141194810 | atenolol+bendroflumethiazide |
|  |  | 1141195254 | triamterene+furosemide 50mg/40mg tablet |
|  |  | 1141195258 | furosemide+potassium 20mg/10mmol m/r tablet |
|  |  | 1141201038 | valsartan+hydrochlorothiazide 80mg/12.5mg tablet |
|  |  | 1141201244 | Eplerenone |
| gp_scripts | Primary care prescription for lipid modifying agents | C10AA | HMG CoA reductase inhibitors (statins) |
|  |  | C10AB | Fibrates |
|  |  | C10AC | Bile acid sequestrants |
|  |  | C10AD | Nicotinic acid and derivatives |
|  |  | C10AX | Other lipid modifying agents |
|  |  | C10BA | Combinations of various lipid modifying agents |
|  |  | C10BX | Lipid modifying agents in combination with other drugs |
| gp_scripts | Primary care prescription for antihypertensives | C02A | Antiadrenergic agents, centrally acting |
|  |  | C02B | Antiadrenergic agents, ganglion-blocking |
|  |  | C02C | Antiadrenergic agents, peripherally acting |
|  |  | C02D | Arteriolar smooth muscle, agents acting on |
|  |  | C02K | Other antihypertensives |
|  |  | C02L | Antihypertensives and diuretics in combination |
|  |  | C02N | Combinations of antihypertensives in ATC-GR. C02 |
|  | Primary care prescription for diuretics | C03A | Low-ceiling diuretics, thiazides |
| gp_scripts |  | C03B | Low-ceiling diuretics, excl. thiazides |
|  |  | C03C | High-ceiling diuretics |
|  |  | C03D | Aldosterone antagonists and other potassium-sparing agents |
|  |  | C03E | Diuretics and potassium-sparing agents in combination |
|  |  | C03X | Other diuretics |
| gp_scripts | Primary care prescription for beta blocking agents | C07A | Beta blocking agents |
|  |  | C07B | Beta blocking agents and thiazides |
|  |  | C07C | Beta blocking agents and other diuretics |
|  |  | C07D | Beta blocking agents, thiazides and other diuretics |
|  |  | C07E | Beta blocking agents and vasodilators |
|  |  | C07F | Beta blocking agents, other combinations |
| gp_scripts | Primary care prescription for calcium channel blockers | C08C | Selective calcium channel blockers with mainly vascular effects |
|  |  | C08D | Selective calcium channel blockers with direct cardiac effects |
|  |  | C08E | Non-selective calcium channel blockers |
|  |  | C08G | Calcium channel blockers and diuretics |
| gp_scripts | Primary care prescription for agents acting on the renin-angiotensin system | C09A | ACE inhibitor, plain |
|  |  | C09B | ACE inhibitor, combinations |
|  |  | C09C | Angiotensin II receptor blockers (ARBs), plain |
|  |  | C09D | Angiotensin II receptor blockers (ARBs), combinations |
|  |  | C09X | Other agents acting on the renin-angiotensin system |

Data field 20003 (treatment/medication code, self-reported) and data codes correspond to UK Biobank main tabular phenotype data. More details can be found on the UKB Showcase ([Data Field 20003](https://biobank.ndph.ox.ac.uk/ukb/field.cgi?id=20003)). gp_scripts correspond to general practice (primary care) prescription data and ATC codes were used to querying.

Supplementary Table 4. List of quantitative traits assessed for carriers and noncarriers

| Category | UK Biobank Data Field | Description |
| --- | --- | --- |
| Physical measures | 48 | Waist circumference |
|  | 49 | Hip circumference |
|  | 4079 | Diastolic Blood Pressure, automated reading |
|  | 4080 | Systolic Blood Pressure, automated reading |
|  | 21001 | Body mass index (BMI) |
|  | 21002 | Weight |
|  | 21021 | Pulse wave Arterial Stiffness index |
| Blood biomarkers | 30600 | Albumin |
|  | 30610 | Alkaline phosphatase |
|  | 30620 | Alanine aminotransferase |
|  | 30630 | Apolipoprotein A |
|  | 30640 | Apolipoprotein B |
|  | 30650 | Aspartate aminotransferase |
|  | 30660 | Direct bilirubin |
|  | 30670 | Urea |
|  | 30680 | Calcium |
|  | 30690 | Cholesterol |
|  | 30700 | Creatinine |
|  | 30710 | C-reactive protein |
|  | 30720 | Cystatin C |
|  | 30730 | Gamma glutamyltransferase |
|  | 30740 | Glucose |
|  | 30750 | Glycated haemoglobin (HbA1c) |
|  | 30760 | HDL cholesterol |
|  | 30770 | IGF-1 |
|  | 30780 | LDL direct |
|  | 30790 | Lipoprotein A |
|  | 30800 | Oestradiol |
|  | 30810 | Phosphate |
|  | 30820 | Rheumatoid factor |
|  | 30830 | SHBG |
|  | 30840 | Total bilirubin |
|  | 30850 | Testosterone |
|  | 30860 | Total protein |
|  | 30870 | Triglycerides |
|  | 30880 | Urate |
|  | 30890 | Vitamin D |
| Hematological traits | 30000 | White blood cell (leukocyte) count |
|  | 30010 | Red blood cell (erythrocyte) count |
|  | 30020 | Haemoglobin concentration |
|  | 30030 | Haematocrit percentage |
|  | 30040 | Mean corpuscular volume |
|  | 30050 | Mean corpuscular haemoglobin |
|  | 30060 | Mean corpuscular haemoglobin concentration |
|  | 30070 | Red blood cell (erythrocyte) distribution width |
|  | 30080 | Platelet count |
|  | 30090 | Platelet crit |
|  | 30100 | Mean platelet (thrombocyte) volume |
|  | 30110 | Platelet distribution width |
|  | 30120 | Lymphocyte count |
|  | 30130 | Monocyte count |
|  | 30140 | Neutrophill count |
|  | 30150 | Eosinophill count |
|  | 30160 | Basophill count |
|  | 30170 | Nucleated red blood cell count |
|  | 30180 | Lymphocyte percentage |
|  | 30190 | Monocyte percentage |
|  | 30200 | Neutrophill percentage |
|  | 30210 | Eosinophill percentage |
|  | 30220 | Basophill percentage |
|  | 30230 | Nucleated red blood cell percentage |
|  | 30240 | Reticulocyte percentage |
|  | 30250 | Reticulocyte count |
|  | 30260 | Mean reticulocyte volume |
|  | 30270 | Mean sphered cell volume |
|  | 30280 | Immature reticulocyte fraction |
|  | 30290 | High light scatter reticulocyte percentage |
|  | 30300 | High light scatter reticulocyte count |
| Urine biomarkers | 30500 | Microalbumin in urine |
|  | 30510 | Creatinine (enzymatic) in urine |
|  | 30520 | Potassium in urine |
|  | 30530 | Sodium in urine |
| Cognition | 20016 | Fluid intelligence score (assessment center touchscreen) |
|  | 20023 | Reaction time: Mean time to correctly identify matches |
|  | 6138, 10722 | Educational qualifications, education qualifications pilot |

Data fields and data codes correspond to UK Biobank main tabular phenotype data. More details can be found on the UKB Showcase ([physical measures](https://biobank.ndph.ox.ac.uk/ukb/label.cgi?id=706), [blood biomarkers](https://biobank.ndph.ox.ac.uk/ukb/label.cgi?id=17518), [hematological traits](https://biobank.ndph.ox.ac.uk/ukb/label.cgi?id=100081), [urine biomarkers](https://biobank.ndph.ox.ac.uk/ukb/label.cgi?id=100083), [cognition](https://biobank.ndph.ox.ac.uk/ukb/label.cgi?id=100026)).

Supplementary Table 5. *APOE* genotype of APOECh carriers and matched noncarriers

| Genotype | rs429358 | rs7412 | APOECh carriers (N=36) | Noncarriers (N=129,240) | Noncarriers with AD (N=660) | Probability of genotype enrichment in carriers | With CVD (carriers/noncarriers) |
| --- | --- | --- | --- | --- | --- | --- | --- |
| e2/e2 | T/T | T/T | 0 | 518 (0.4%) | 1 (0.2%) | 0.87 | 0/196 |
| e2/e3 | T/T | C/T | 1 (2.8%) | 15,887 (12.3%) | 28 (4.2%) | 0.99 | 0/8,586 |
| e2/e4 or e1/e3 | C/T | C/T | 0 | 3,216 (2.5%) | 18 (2.7%) | 0.40 | 0/1,743 |
| e3/e3 | T/T | C/C | 30 (83.3%) | 75,200 (58.2%) | 209 (31.7%) | 0.001 | 19/42,353 |
| e3/e4 | C/T | C/C | 5 (13.9%) | 30,781 (23.8%) | 302 (45.8%) | 0.95 | 3/17,459 |
| e4/e4 | C/C | C/C | 0 | 2,785 (2.2%) | 92 (13.9%) | 0.46 | 0/1,505 |
| NA | Missing | | 0 | 853 (0.7%) | 10 (1.5%) | 0.79 | 0/474 |

Breakdown of *APOE* genotype among APOECh carriers and matched noncarriers as well as the number of individuals with one or more diagnosis in cardiovascular disease.

Supplementary Table 6. Screening APOECh carriers for *APOE* protective variants and mutations reported for neurodegenerative disorders with a Mendelian inheritance

| Gene | SNPs assessed | SNPs found in UKB data | APOECh carriers | APOECh noncarriers | Additional annotations |
| --- | --- | --- | --- | --- | --- |
| APOE | rs199768005  rs267606661 | rs199768005  rs267606661 | None | rs199768005: 23 individuals with 1 copy  rs267606661: 17 individuals with 1 copy  No individuals with both variants | *APOE* rs199768005-A is also known as V236E Jacksonville variant  rs267606661-G is also known as R251G  Both rare variants are protective for AD (17). |
| APP | rs63750264  rs63749964  rs63750671  rs63750066  rs63750399  rs63750734  rs63751039  rs63750973  rs63750643  rs193922916  rs63750847 | rs63750264  rs63750066  rs63750847 | None | rs63750066: 6 individuals with 1 copies of risk allele T | *APP* rs63750066 allele T is associated with familial AD (18, 19). |
| PSEN1 | rs63750306  rs63750590  rs63750526  rs63751235  rs661  rs63751037  rs63749885  rs63750231  rs63751229  rs63751272  rs63751223  rs63750391  rs63751163  rs63749891  rs281875357  rs63751141  rs63750082  rs121917807  rs63750265  rs63751144  rs63750886  rs121917808  rs63750599  rs63750083  rs63749824  rs63750577  rs267606983  rs63750687 | rs63750526  rs63749824 | None | rs63749824: 5 individuals with 1 copy of risk allele T  rs63750526: 1 individual with 1 copy of risk allele A | *PSEN1* rs63749824 allele T is associated with late-onset AD in a carrier; unaffected mutation carrier has increased CSF Aβ (20). Variant rs63750526 allele A is associated with early-onset AD (21). |
| PSEN2 | rs63750215  rs28936379  rs63750110  rs63750666  rs63749851  rs63749884  rs28936380  rs63750197  rs63750048 | rs63750110  rs63750666  rs63750197  rs63750048 | None | rs63750048: 1 individual with 1 copy of risk allele T  rs63750197: 427 individuals with 1 copy of risk allele T  rs63750666: 4 individuals with 1 copy of risk allele T  rs63750110: 55 individuals with 1 copy of risk allele C | *PSEN2* rs63750048 allele T is associated with familial AD as well as parkinsonism and Lewy body dementia (22).  Variant rs63750197 was reported in association with AD (23), but ClinVar shows that allele T is benign. Variant rs63750110 allele C is associated with early onset AD in an individual with APOE2/3 genotype (24). Variant rs63750110 allele C is associated with Alzheimer’s disease (24). |
| GRN | rs63750077  rs63751006  rs63750331  rs606231220  rs63749801  rs606231221  rs63751243  rs63751294  rs63749905  rs63751085  rs63749877  rs5848 | rs63751294  rs5848 | rs5848: 4 individuals with 1 copy of risk allele T; 1 individual with 2 copies of risk allele T | rs63751294: 5 individuals with 1 copy of risk allele T  rs5848: 2934 individuals with 2 copies of risk allele T | *GRN* rs63751294 allele T is associated with ubiquitin-positive frontotemporal lobar degeneration (FTLD) (25, 26). Variant rs5848 showed significant increase in T/T genotype among FTLD (27). This variant was only found in the 140k WGS data. |
| SORT1 | rs12740374 | rs12740374  rs17646665 | rs12740374: 1 individual with 1 copy of risk allele T; 2 individuals with 2 copies of risk allele T  rs17646665: 1 individual with 1 copy of protective allele G | rs12740374: 13668 individuals with 1 copy of risk allele T; 1927 individuals with 2 copies of risk allele T  rs17646665: 2702 individuals with 1 copy of protective allele G; 1926 individuals with 2 copies of protective allele G | *SORT1* rs12740374 allele T creates a transcription factor binding site and results increased SORT1 expression (28), which in turn controls PGRN levels and plays a role in the lysosomal pathway (29). Variant rs17646665 allele G is significantly associated with reduced risk of AD (30). |
| MAPT | rs63751273  rs63750376  rs63750424  rs63750972  rs63750308  rs63751011  rs1568327531  rs63750570  rs63750756  rs63751165  rs63750512  rs63751438  rs63750912  rs63750711  rs63750129  rs63751264  rs63750959  rs63750635  rs63751394  rs63751392  rs63750349  rs63750425  rs63750092  rs63751391 | rs63750424  rs63751011  rs63750512  rs63750129  rs63750959  rs63750425 | None | rs63751011: 2 individuals with 1 copy of risk allele T  rs63750424: 6 individual with 1 copy of risk allele T | *MAPT* rs63751011 allele T is associated with frontotemporal dementia (31-36).  Variant rs63750424 has been associated with frontotemporal dementia (31, 37) and has been reported to clinically resembles AD (38). |
| APBB2 | rs13133980 | rs13133980 | rs13133980: 2 individuals with 1 copy of risk allele G; 3 individuals with 2 copies of risk allele G | rs13133980: 19,227 individuals with 1 copy of risk allele G; 7,258 individuals with 2 copies of risk allele G | *APBB2* rs13133980 allele G showed stronger association with age of AD onset before 75 years of age (39). However, another study did not replicate this finding and rs13133980 was not associated with AD regardless of age of onset or APOE status (40). |

We screened APOECh carriers and matched noncarriers for rare protective variants in APOE (Jacksonville and R251G) and mutations reported for neurodegenerative disorders with a Mendelian inheritance (*APP*, *PSEN1*, *PSEN2*, *GRN*, *SORT1*, *MAPT*, and *ABB2*).

Supplementary Table 7. Empirical p-values of binary traits

| Trait | APOECh carriers with event (N=36) | Noncarriers with event (N=129,240) | P-value |
| --- | --- | --- | --- |
| AD (ICD + self-report) | 0 | 660 (0.51%) | 0.83 |
| Parental history of AD (self-report) | 4 (11.1%) | 17428 (13.5%) | 0.74 |
| MCI or other cognitive function symptoms | 0 | 755 (0.58%) | 0.81 |
| CVD (ICD + self-report) | 22 (61.1%) | 72652 (56.2%) | 0.34 |
| Dyslipidemia (ICD + self-report) | 10 (27.8%) | 24643 (19.1%) | 0.13 |
| Primary hypertension (ICD + self-report) | 15 (41.7%) | 44869 (34.7%) | 0.24 |
| Self-reported statin at baseline | 5 (13.9%) | 15970 (12.3%) | 0.46 |
| Prescription for lipid-lowering drugs | 6 (35.3%) | 16436 (12.7%) | 0.30 |
| Use of lipid-lowering drugs (prescription + self-report) | 9 (25%) | 27281 (21.1%) | 0.34 |
| Prescription for antihypertensive drugs | 9 (52.9%) | 22011 (17.0%) | 0.15 |
| Use of antihypertensive (prescription + self-report) | 12 (33.3%) | 36029 (27.9%) | 0.29 |
| MACE (ICD + self-report) | 4 (11.1%) | 10495 (8.12%) | 0.33 |
| Type 2 diabetes (ICD + self-report) | 1 (2.8%) | 8511 (6.5%) | 0.91 |
| Cerebral amyloid angiopathy (ICD) | 0 | 22 (0.02%) | 0.99 |
| Vascular dementia (ICD) | 0 | 295 (0.23%) | 0.92 |
| Parkinson’s disease (ICD + self-report) | 1 (2.8%) | 719 (0.56%) | 0.18 |
| Depression (ICD + self-report) | 5 (14%) | 9382 (7.26%) | 0.12 |
| Schizophrenia (ICD + self-report) | 0 | 294 (0.23%) | 0.92 |
| Bipolar disorder (ICD + self-report) | 1 (2.8%) | 677 (0.52%) | 0.17 |

We compared whether carrier event frequency is higher or lower than expected based on noncarrier prevalence for selected binary traits. Definitions for these conditions can be found under Phenotype definitions section in Supplementary Methods and in Supplementary Tables 2 and 3.

Supplementary Table 8. Kolmogorov-Smirnov test of quantitative traits and PRS among all 36 carriers and matching controls

| Category | UKB Data Field | Median of carriers  N=36 | Median of noncarriers  N=129,240 | KS test |
| --- | --- | --- | --- | --- |
| Lipid biomarkers | Apolipoprotein_A_30630 | 1.64 | 1.55 | D=0.19 P-value=0.239 |
|  | Apolipoprotein_B_30640 | 0.88 | 1.02 | D=0.31 P-value=0.004 |
|  | statin_adj_Apolipoprotein_B_30640 | 0.94 | 1.06 | D=0.25 P-value=0.036 |
|  | C-Reactive_protein_30710 | 1.12 | 1.32 | D=0.12 P-value=0.662 |
|  | Cholesterol_30690 | 5.32 | 5.73 | D=0.18 P-value=0.263 |
|  | statin_adj_Cholesterol_30690 | 5.80 | 5.91 | D=0.15 P-value=0.427 |
|  | HDL_30760 | 1.56 | 1.47 | D=0.2 P-value=0.225 |
|  | LDL_30780 | 3.25 | 3.55 | D=0.23 P-value=0.072 |
|  | statin_adj_LDL_30780 | 3.36 | 3.70 | D=0.21 P-value=0.119 |
|  | Lipoprotein_A_30790 | 22.00 | 20.50 | D=0.11 P-value=0.838 |
|  | Triglyceride_30870 | 1.71 | 1.42 | D=0.16 P-value=0.329 |
| Physical measures | DBP_4079 | 83.00 | 81.00 | D=0.1 P-value=0.726 |
|  | adjusted_DBP_4079 | 83.25 | 83.00 | D=0.1 P-value=0.806 |
|  | SBP_4080 | 129.50 | 136.00 | D=0.15 P-value=0.332 |
|  | adjusted_SBP_4080 | 136.25 | 137.00 | D=0.11 P-value=0.755 |
|  | Pulse_wave_Arterial_Stiffness_index_21021 | 10.19 | 8.78 | D=0.38 P-value=0.149 |
|  | Waist_circumference_48 | 86.00 | 87.00 | D=0.1 P-value=0.694 |
|  | Hip_circumference_49 | 102.00 | 102.00 | D=0.13 P-value=0.411 |
|  | Body_mass_index_21001 | 25.65 | 26.47 | D=0.16 P-value=0.3 |
|  | Weight_21002 | 71.70 | 73.50 | D=0.15 P-value=0.375 |
| Urine biomarkers | Microalbumin_30500 | 7.80 | 11.20 | D=0.54 P-value=0.02 |
|  | Creatinine_30510 | 8737.00 | 6792.00 | D=0.14 P-value=0.48 |
|  | Potassium_30520 | 58.60 | 55.00 | D=0.08 P-value=0.957 |
|  | Sodium_30530 | 60.20 | 63.70 | D=0.08 P-value=0.972 |
| Blood biomarkers | Albumin_30600 | 44.86 | 45.14 | D=0.16 P-value=0.444 |
|  | Alkaline_phosphatase_30610 | 84.10 | 80.30 | D=0.16 P-value=0.327 |
|  | Alanine_aminotransferase_30620 | 20.52 | 18.94 | D=0.14 P-value=0.516 |
|  | Aspartate_aminotransferase_30650 | 26.70 | 23.70 | D=0.25 P-value=0.029 |
|  | Direct_bilirubin_30660 | 1.42 | 1.55 | D=0.16 P-value=0.411 |
|  | Urea_30670 | 4.90 | 5.17 | D=0.12 P-value=0.702 |
|  | Calcium_30680 | 2.39 | 2.38 | D=0.16 P-value=0.494 |
|  | Creatinine_30700 | 69.90 | 66.90 | D=0.17 P-value=0.288 |
|  | Cystatin_C_30720 | 0.86 | 0.87 | D=0.1 P-value=0.887 |
|  | Gamma_glutamyltransferase_30730 | 25.60 | 23.90 | D=0.13 P-value=0.624 |
|  | Glucose_30740 | 4.71 | 4.91 | D=0.25 P-value=0.067 |
|  | HbA1c_30750 | 35.00 | 35.00 | D=0.12 P-value=0.609 |
|  | IGF-1_30770 | 20.47 | 21.17 | D=0.17 P-value=0.29 |
|  | Oestradiol_30800 | 396.40 | 366.20 | D=0.26 P-value=0.585 |
|  | Phosphate_30810 | 1.22 | 1.17 | D=0.13 P-value=0.685 |
|  | Rheumatoid_factor_30820 | 12.65 | 16.50 | D=0.65 P-value=0.239 |
|  | SHBG_30830 | 49.88 | 49.66 | D=0.12 P-value=0.821 |
|  | Total_bilirubin_30840 | 7.60 | 7.70 | D=0.07 P-value=0.996 |
|  | Testosterone_30850 | 1.68 | 1.34 | D=0.13 P-value=0.72 |
|  | Total_protein_30860 | 71.92 | 72.15 | D=0.12 P-value=0.777 |
|  | Urate_30880 | 303.40 | 284.20 | D=0.16 P-value=0.338 |
|  | Vitamin D_30890 | 56.80 | 47.30 | D=0.21 P-value=0.115 |
| Hematological traits | Leukocyte_count_30000 | 6.36 | 6.65 | D=0.12 P-value=0.691 |
|  | Erythrocyte_count_30010 | 4.50 | 4.41 | D=0.13 P-value=0.543 |
|  | Haemoglobin_concentration_30020 | 13.70 | 13.86 | D=0.12 P-value=0.645 |
|  | Haematrocrit_percentage_30030 | 40.51 | 40.20 | D=0.11 P-value=0.807 |
|  | Mean_corpuscular_volume_30040 | 90.85 | 91.29 | D=0.07 P-value=0.995 |
|  | Mean_corpuscular_haemoglobin_30050 | 31.47 | 31.50 | D=0.12 P-value=0.675 |
|  | Mean_corpuscular_haemoglobin_concentration_30060 | 34.19 | 34.45 | D=0.15 P-value=0.404 |
|  | Erythrocyte_distribution width_30070 | 13.50 | 13.31 | D=0.23 P-value=0.038 |
|  | Platelet_count_30080 | 255.00 | 254.00 | D=0.11 P-value=0.78 |
|  | Platelet crit_30090 | 0.25 | 0.24 | D=0.18 P-value=0.195 |
|  | Thrombocyte_volume_30100 | 9.60 | 9.20 | D=0.26 P-value=0.017 |
|  | Platelet_distribution_width_30110 | 16.48 | 16.40 | D=0.13 P-value=0.537 |
|  | Lymphocyte_count_30120 | 1.80 | 1.89 | D=0.22 P-value=0.067 |
|  | Monocyte_count_30130 | 0.42 | 0.43 | D=0.08 P-value=0.924 |
|  | Neutrophill_count_30140 | 4.09 | 4.03 | D=0.09 P-value=0.891 |
|  | Eosinophill_count_30150 | 0.13 | 0.13 | D=0.09 P-value=0.814 |
|  | Basophill_count_30160 | 0.02 | 0.02 | D=0.1 P-value=0.599 |
|  | Nucleated_red_blood_cell_count_30170 | 0.00 | 0.00 | D=0.01 P-value=1 |
|  | Lymphocyte_percentage_30180 | 26.20 | 28.68 | D=0.19 P-value=0.142 |
|  | Monocyte_percentage_30190 | 6.48 | 6.68 | D=0.13 P-value=0.615 |
|  | Neutrophill_percentage_30200 | 61.70 | 61.30 | D=0.18 P-value=0.18 |
|  | Eosinophill_percentage_30210 | 1.80 | 2.08 | D=0.1 P-value=0.841 |
|  | Basophill_percentage_30220 | 0.40 | 0.43 | D=0.14 P-value=0.466 |
|  | Nucleated_red_blood_cell_percentage_30230 | 0.00 | 0.00 | D=0.01 P-value=1 |
|  | Reticulocyte_percentage_30240 | 1.09 | 1.25 | D=0.2 P-value=0.126 |
|  | Reticulocyte_count_30250 | 0.05 | 0.06 | D=0.18 P-value=0.183 |
|  | Mean_reticulocyte_volume_30260 | 107.42 | 105.61 | D=0.14 P-value=0.451 |
|  | Mean_sphered_cell_volume_30270 | 84.30 | 82.70 | D=0.16 P-value=0.333 |
|  | Immature_reticulocyte_fraction_30280 | 0.27 | 0.29 | D=0.22 P-value=0.053 |
|  | High_light_scatter_reticulocyte_percentage_30290 | 0.27 | 0.36 | D=0.22 P-value=0.073 |
|  | High_light_scatter_reticulocyte_count_30300 | 0.01 | 0.02 | D=0.23 P-value=0.03 |
| Cognition | education_years | 6 | 6 | D=0.24 P-value=0.316 |
|  | fluid_intelligence_score_center_20016 | 522 | 535 | D=0.07 P-value=0.98 |
|  | reaction_time_20023 | 14 | 15 | D=0.11 P-value=0.436 |
| Dyslipidemia PRS | HDL_26242 | -0.04 | 0.01 | D=0.13 P-value=0.53 |
|  | LDL_26250 | -0.11 | -0.06 | D=0.1 P-value=0.79 |
| Cardiovascular PRS | Atrial_fibrillation_26212 | 0.33 | 0.10 | D=0.12 P-value=0.58 |
|  | Cardiovascular_disease_26223 | -0.35 | -0.10 | D=0.16 P-value=0.29 |
|  | Coronary_artery_disease_26227 | -0.31 | -0.16 | D=0.14 P-value=0.48 |
|  | Hypertension_26244 | 0.09 | -0.04 | D=0.15 P-value=0.34 |
|  | Stroke_26248 | -0.04 | -0.02 | D=0.08 P-value=0.96 |
| Neurological disease PRS | Alzheimer’s_disease_26206 | -0.31 | -0.08 | D=0.25 P-value=0.02 |
|  | Multiple_sclerosis_26254 | 0.06 | -0.15 | D=0.15 P-value=0.33 |
|  | Parkinson’s_disease_26260 | -0.54 | -0.17 | D=0.21 P-value=0.06 |

For quantitative traits, a two-sided Kolmogorov-Smirnov (KS) test was used to test whether the distributions for carriers and noncarriers differed significantly. Definitions for these conditions can be found in Supplementary Table 4. Polygenic risk scores (PRS) were generated by the UK Biobank and only standard PRS (derived without UKB samples) were used.

Supplementary Table 9. Mendelian randomization of statin-adjusted apoB and AD

| **Exposure** | **Outcome** | **Method** | **NSNP** | **Beta** | **SE** | **P** | **FDR** | **Q** | **Q_df** | **Q_pval** | **Egger intercept (SE)** | **Egger intercept p-value** |
| --- | --- | --- | --- | --- | --- | --- | --- | --- | --- | --- | --- | --- |
| Sinnott-Armstrong apoB | Bellenguez AD | MR Egger | 991 | 0.377 | 0.022 | 3.79E-56 | 1.51E-55 | 9731.751 | 990 | p<2.23e-308 | -0.013 (0.002) | 9.92E-16 |
| Sinnott-Armstrong apoB | Bellenguez AD | Inverse variance weighted | 991 | 0.254 | 0.017 | 7.49E-50 | 1.50E-49 | 9117.519 | 989 | p<2.23e-308 | NA | NA |
| Sinnott-Armstrong apoB | Bellenguez AD | Weighted mode | 991 | -0.061 | 0.017 | 2.66E-04 | 3.55E-04 | NA | NA | NA | NA | NA |
| Sinnott-Armstrong apoB | Bellenguez AD | Weighted median | 991 | 0.011 | 0.014 | 0.430 | 0.430 | NA | NA | NA | NA | NA |
| **APOE removed (hg38: 19:44905796-44909393)** | | | | | | | | | | | | |
| Sinnott-Armstrong apoB | Bellenguez AD | MR Egger | 765 | -0.083 | 0.014 | 5.21E-09 | 6.95E-09 | 1245.573 | 763 | 8.23E-26 | 1.30E-03 (7.67E-04) | 0.091 |
| Sinnott-Armstrong apoB | Bellenguez AD | Inverse variance weighted | 765 | -0.065 | 0.010 | 7.37E-12 | 1.47E-11 | 1250.237 | 764 | 4.23E-26 | NA | NA |
| Sinnott-Armstrong apoB | Bellenguez AD | Weighted mode | 765 | -0.063 | 0.016 | 9.16E-05 | 9.16E-05 | NA | NA | NA | NA | NA |
| Sinnott-Armstrong apoB | Bellenguez AD | Weighted median | 765 | -0.073 | 0.010 | 1.55E-12 | 6.21E-12 | NA | NA | NA | NA | NA |

Four Mendelian randomization (MR) approaches were assessed: MR-Egger, inverse variance weighted, weighted mode, and weighted median. The *APOE* region was further removed as sensitivity analysis.

Abbreviations:

NSNP: number of SNPs | SE: standard error | P: p-value of MR analysis | FDR: false discovery rate of MR analysis | Q: heterogeneity statistic that provides evidence for heterogeneity and invalid instruments | Q_df: degrees of freedom (number of instrumental variables minus 1) | Q_pval: p-value of the heterogeneity statistic

Supplementary Table 10. Availability and overlap of APOECh carriers with brain imaging, metabolomics, and proteomics data

|  | Brain MRI completed | Metabolomics | Proteomics |
| --- | --- | --- | --- |
| Brain MRI completed | 3 | 1 | 1 |
| Metabolomics |  | 9 | 2 |
| Proteomics |  |  | 7 |

Although multiple data modalities are available in the UKB, there is little overlap in the number of APOECh carriers with more than two types of data.

Supplementary Table 11. Summary of phenotypic data availability for carriers and noncarriers

|  | All APOECh carriers (N=37) | APOECh EUR carriers (N=36) | Matched EUR noncarriers (N=129,240) | P-value for differences in EUR data availability |
| --- | --- | --- | --- | --- |
| ≥ 1 ICD-9 or ICD-10 code | 30 (81.1%) | 30 (83.3%) | 113,386 (87.7%) | 0.85 |
| ≥ 1 Self-reported non-cancer illness(es) | 24 (64.7%) | 23 (63.9%) | 98,938 (76.6%) | 0.97 |
| ≥ 1 Self-reported medication(s) | 30 (81.1%) | 29 (80.6%) | 97,146 (75.2%) | 0.30 |
| Primary care data available | 18 (48.6%) | 17 (47.2%) | 60,680 (46.9%) | 0.55 |
| Primary care prescription data available | 18 (48.6%) | 17 (47.2%) | 58,575 (45.3%) | 0.47 |
| ≥ 1 lipid biomarker measurement | 32 (86.5%) | 31 (86.1%) | 123,587 (95.6%) | 0.995 |

Within a given individual, the amount of phenotypic data available is variable. We assessed whether there are significant differences in the phenotype data that are available for APOECh carriers and matched noncarriers.

Supplementary Table 12. Linear and logistic regression of HDL, LDL, CVD, and AD with PRS as a covariate between APOECh carriers and noncarriers

| Trait | PRS UKB Data Field | Cases in carriers (N=36) | Cases in noncarriers (N=129,240) | Slope of carrier status | P-value of carrier status |
| --- | --- | --- | --- | --- | --- |
| HDL | 26242 | NA | NA | -0.03 | 0.64 |
| LDL | 26250 | NA | NA | -0.21 | 0.16 |
| CVD | 26223 | 22 | 72,651 | 0.22 | 0.52 |
| AD | 26206 | 0 | 660 | 0.69 | 0.95 |

In addition to comparing the distributions of PRS for HDL and LDL and polygenic risk for CVD and AD in APOECh carriers vs. matched noncarriers, we assessed potential differences by carrier status using linear and logistic regressions accounting for PRS as a covariate.

**Supplementary Figures**

Supplementary Figure 1. Selection of APOE Christchurch carriers and matched controls from the UK Biobank


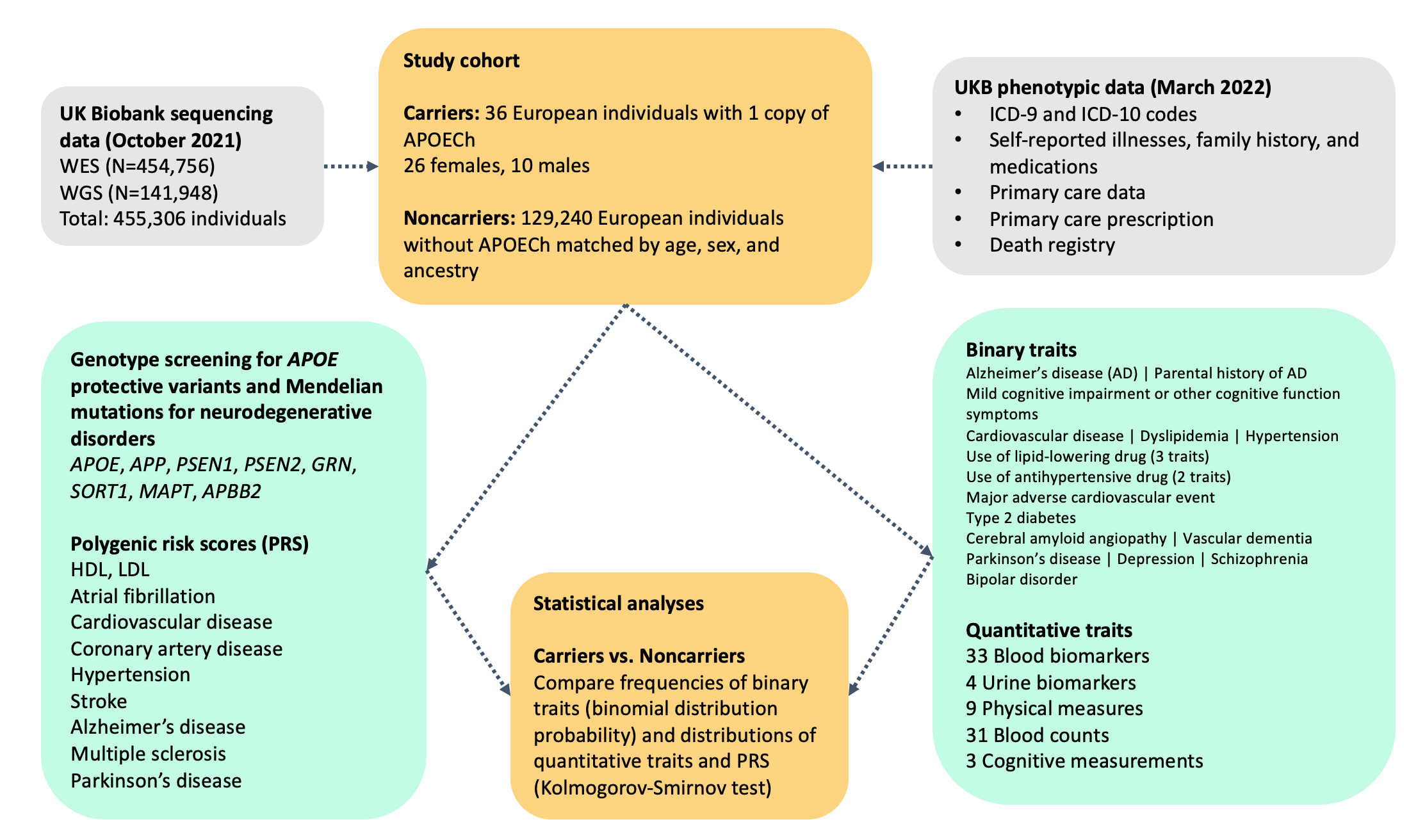


This schematic demonstrates the design of this study. We identified 36 European individuals with 1 copy of the rare APOE Christchurch variant and examined the disease and biomarker profiles of APOECh carriers vs. matched noncarriers.

Supplementary Figure 2. Cumulative distributions of physical measures and Kolmogorov-Smirnov test results

Supplementary Figure 3. Cumulative distributions of urine biomarkers and Kolmogorov-Smirnov test results

Supplementary Figure 4. Cumulative distributions of blood biomarkers and Kolmogorov-Smirnov test results

Supplementary Figure 5. Cumulative distributions of hematological traits and Kolmogorov-Smirnov test results

Supplementary Figure 6. Cumulative distributions of lipid biomarkers and Kolmogorov-Smirnov test results

**Supplementary References**

1. Bycroft C, Freeman C, Petkova D, Band G, Elliott LT, Sharp K, et al. The UK Biobank resource with deep phenotyping and genomic data. Nature. 2018;562(7726):203-9.

2. Backman JD, Li AH, Marcketta A, Sun D, Mbatchou J, Kessler MD, et al. Exome sequencing and analysis of 454,787 UK Biobank participants. Nature. 2021;599(7886):628-34.

3. Halldorsson BV, Eggertsson HP, Moore KHS, Hauswedell H, Eiriksson O, Ulfarsson MO, et al. The sequences of 150,119 genomes in the UK Biobank. Nature. 2022;607(7920):732-40.

4. Szustakowski JD, Balasubramanian S, Kvikstad E, Khalid S, Bronson PG, Sasson A, et al. Advancing human genetics research and drug discovery through exome sequencing of the UK Biobank. Nat Genet. 2021;53(7):942-8.

5. Sinnott-Armstrong N, Tanigawa Y, Amar D, Mars N, Benner C, Aguirre M, et al. Genetics of 35 blood and urine biomarkers in the UK Biobank. Nat Genet. 2021;53(2):185-94.

6. Evangelou E, Warren HR, Mosen-Ansorena D, Mifsud B, Pazoki R, Gao H, et al. Genetic analysis of over 1 million people identifies 535 new loci associated with blood pressure traits. Nat Genet. 2018;50(10):1412-25.

7. Cheesman R, Coleman J, Rayner C, Purves KL, Morneau-Vaillancourt G, Glanville K, et al. Familial Influences on Neuroticism and Education in the UK Biobank. Behav Genet. 2020;50(2):84-93.

8. Thompson DJ, Wells D, Selzam S, Peneva I, Moore R, Sharp K, et al. UK Biobank release and systematic evaluation of optimised polygenic risk scores for 53 diseases and quantitative traits. 2022.

9. Pan-UKB [Available from: <https://pan.ukbb.broadinstitute.org>.

10. Bellenguez C, Kucukali F, Jansen IE, Kleineidam L, Moreno-Grau S, Amin N, et al. New insights into the genetic etiology of Alzheimer's disease and related dementias. Nat Genet. 2022;54(4):412-36.

11. Ferkingstad E, Sulem P, Atlason BA, Sveinbjornsson G, Magnusson MI, Styrmisdottir EL, et al. Large-scale integration of the plasma proteome with genetics and disease. Nat Genet. 2021;53(12):1712-21.

12. Wingo TS, Cutler DJ, Wingo AP, Le NA, Rabinovici GD, Miller BL, et al. Association of Early-Onset Alzheimer Disease With Elevated Low-Density Lipoprotein Cholesterol Levels and Rare Genetic Coding Variants of APOB. JAMA Neurol. 2019;76(7):809-17.

13. Picard C, Nilsson N, Labonte A, Auld D, Rosa-Neto P, Alzheimer's Disease Neuroimaging I, et al. Apolipoprotein B is a novel marker for early tau pathology in Alzheimer's disease. Alzheimers Dement. 2022;18(5):875-87.

14. Takechi R, Galloway S, Pallebage-Gamarallage MM, Wellington CL, Johnsen RD, Dhaliwal SS, et al. Differential effects of dietary fatty acids on the cerebral distribution of plasma-derived apo B lipoproteins with amyloid-beta. Br J Nutr. 2010;103(5):652-62.

15. Martins IJ, Berger T, Sharman MJ, Verdile G, Fuller SJ, Martins RN. Cholesterol metabolism and transport in the pathogenesis of Alzheimer's disease. J Neurochem. 2009;111(6):1275-308.

16. Bereczki E, Bernat G, Csont T, Ferdinandy P, Scheich H, Santha M. Overexpression of human apolipoprotein B-100 induces severe neurodegeneration in transgenic mice. J Proteome Res. 2008;7(6):2246-52.

17. Le Guen Y, Belloy ME, Grenier-Boley B, de Rojas I, Castillo-Morales A, Jansen I, et al. Association of Rare APOE Missense Variants V236E and R251G With Risk of Alzheimer Disease. JAMA Neurol. 2022;79(7):652-63.

18. Armstrong J, Boada M, Rey MJ, Vidal N, Ferrer I. Familial Alzheimer disease associated with A713T mutation in APP. Neurosci Lett. 2004;370(2-3):241-3.

19. Rossi G, Giaccone G, Maletta R, Morbin M, Capobianco R, Mangieri M, et al. A family with Alzheimer disease and strokes associated with A713T mutation of the APP gene. Neurology. 2004;63(5):910-2.

20. Kauwe JS, Jacquart S, Chakraverty S, Wang J, Mayo K, Fagan AM, et al. Extreme cerebrospinal fluid amyloid beta levels identify family with late-onset Alzheimer's disease presenilin 1 mutation. Ann Neurol. 2007;61(5):446-53.

21. Sherrington R, Rogaev EI, Liang Y, Rogaeva EA, Levesque G, Ikeda M, et al. Cloning of a gene bearing missense mutations in early-onset familial Alzheimer's disease. Nature. 1995;375(6534):754-60.

22. Piscopo P, Marcon G, Piras MR, Crestini A, Campeggi LM, Deiana E, et al. A novel PSEN2 mutation associated with a peculiar phenotype. Neurology. 2008;70(17):1549-54.

23. Tedde A, Nacmias B, Ciantelli M, Forleo P, Cellini E, Bagnoli S, et al. Identification of new presenilin gene mutations in early-onset familial Alzheimer disease. Arch Neurol. 2003;60(11):1541-4.

24. Lleo A, Blesa R, Gendre J, Castellvi M, Pastor P, Queralt R, et al. A novel presenilin 2 gene mutation (D439A) in a patient with early-onset Alzheimer's disease. Neurology. 2001;57(10):1926-8.

25. Huey ED, Grafman J, Wassermann EM, Pietrini P, Tierney MC, Ghetti B, et al. Characteristics of frontotemporal dementia patients with a Progranulin mutation. Ann Neurol. 2006;60(3):374-80.

26. Mesulam M, Johnson N, Krefft TA, Gass JM, Cannon AD, Adamson JL, et al. Progranulin mutations in primary progressive aphasia: the PPA1 and PPA3 families. Arch Neurol. 2007;64(1):43-7.

27. Rademakers R, Eriksen JL, Baker M, Robinson T, Ahmed Z, Lincoln SJ, et al. Common variation in the miR-659 binding-site of GRN is a major risk factor for TDP43-positive frontotemporal dementia. Hum Mol Genet. 2008;17(23):3631-42.

28. Musunuru K, Strong A, Frank-Kamenetsky M, Lee NE, Ahfeldt T, Sachs KV, et al. From noncoding variant to phenotype via SORT1 at the 1p13 cholesterol locus. Nature. 2010;466(7307):714-9.

29. Hu F, Padukkavidana T, Vaegter CB, Brady OA, Zheng Y, Mackenzie IR, et al. Sortilin-mediated endocytosis determines levels of the frontotemporal dementia protein, progranulin. Neuron. 2010;68(4):654-67.

30. Andersson CH, Hansson O, Minthon L, Andreasen N, Blennow K, Zetterberg H, et al. A Genetic Variant of the Sortilin 1 Gene is Associated with Reduced Risk of Alzheimer's Disease. J Alzheimers Dis. 2016;53(4):1353-63.

31. Hutton M, Lendon CL, Rizzu P, Baker M, Froelich S, Houlden H, et al. Association of missense and 5'-splice-site mutations in tau with the inherited dementia FTDP-17. Nature. 1998;393(6686):702-5.

32. Goedert M, Spillantini MG, Crowther RA, Chen SG, Parchi P, Tabaton M, et al. Tau gene mutation in familial progressive subcortical gliosis. Nat Med. 1999;5(4):454-7.

33. Janssen JC, Warrington EK, Morris HR, Lantos P, Brown J, Revesz T, et al. Clinical features of frontotemporal dementia due to the intronic tau 10(+16) mutation. Neurology. 2002;58(8):1161-8.

34. Pickering-Brown SM, Richardson AM, Snowden JS, McDonagh AM, Burns A, Braude W, et al. Inherited frontotemporal dementia in nine British families associated with intronic mutations in the tau gene. Brain. 2002;125(Pt 4):732-51.

35. Doran M, du Plessis DG, Ghadiali EJ, Mann DM, Pickering-Brown S, Larner AJ. Familial early-onset dementia with tau intron 10 + 16 mutation with clinical features similar to those of Alzheimer disease. Arch Neurol. 2007;64(10):1535-9.

36. Colombo R, Tavian D, Baker MC, Richardson AM, Snowden JS, Neary D, et al. Recent origin and spread of a common Welsh MAPT splice mutation causing frontotemporal lobar degeneration. Neurogenetics. 2009;10(4):313-8.

37. Reed LA, Grabowski TJ, Schmidt ML, Morris JC, Goate A, Solodkin A, et al. Autosomal dominant dementia with widespread neurofibrillary tangles. Ann Neurol. 1997;42(4):564-72.

38. Rademakers R, Dermaut B, Peeters K, Cruts M, Heutink P, Goate A, et al. Tau (MAPT) mutation Arg406Trp presenting clinically with Alzheimer disease does not share a common founder in Western Europe. Hum Mutat. 2003;22(5):409-11.

39. Li Y, Hollingworth P, Moore P, Foy C, Archer N, Powell J, et al. Genetic association of the APP binding protein 2 gene (APBB2) with late onset Alzheimer disease. Hum Mutat. 2005;25(3):270-7.

40. Golanska E, Sieruta M, Gresner SM, Hulas-Bigoszewska K, Corder EH, Styczynska M, et al. Analysis of APBB2 gene polymorphisms in sporadic Alzheimer's disease. Neurosci Lett. 2008;447(2-3):164-6.
